# Concurrent electrophysiological recording and cognitive testing in a rodent touchscreen environment

**DOI:** 10.1038/s41598-021-91091-9

**Published:** 2021-06-03

**Authors:** Brian D. Kangas, Ann M. Iturra-Mena, Mykel A. Robble, Oanh T. Luc, David Potter, Stefanie Nickels, Jack Bergman, William A. Carlezon, Diego A. Pizzagalli

**Affiliations:** Harvard Medical School, McLean Hospital, 115 Mill Street, Belmont, MA 02478 USA

**Keywords:** Cognitive control, Neurophysiology

## Abstract

Challenges in therapeutics development for neuropsychiatric disorders can be attributed, in part, to a paucity of translational models capable of capturing relevant phenotypes across clinical populations and laboratory animals. Touch-sensitive procedures are increasingly used to develop innovative animal models that better align with testing conditions used in human participants. In addition, advances in electrophysiological techniques have identified neurophysiological signatures associated with characteristics of neuropsychiatric illness. The present studies integrated these methodologies by developing a rat flanker task with electrophysiological recordings based on reverse-translated protocols used in human electroencephalogram (EEG) studies of cognitive control. Various touchscreen-based stimuli were evaluated for their ability to efficiently gain stimulus control and advance to flanker test sessions. Optimized stimuli were then examined for their elicitation of prototypical visual evoked potentials (VEPs) across local field potential (LFP) wires and EEG skull screws. Of the stimuli evaluated, purple and green photographic stimuli were associated with efficient training and expected flanker interference effects. Orderly stimulus-locked outcomes were also observed in VEPs across LFP and EEG recordings. These studies along with others verify the feasibility of concurrent electrophysiological recordings and rodent touchscreen-based cognitive testing and encourage future use of this integrated approach in therapeutics development.

## Introduction

The development of new treatments for neuropsychiatric illness has been challenging^1^. This is due, in part, to a paucity of animal models that quantify translationally-relevant aspects of complex behavioral processes and has highlighted the need for innovative animal models to better assay behavioral phenotypes implicated in these disorders^2,3^. Use of methods involving touch-sensitive apparatus is an increasingly popular means to develop and empirically validate tasks to model complex behavioral processes and cognitive function^4^. Indeed, touchscreen chambers provide an extremely versatile means to expose laboratory animals to (1) traditional models of animal cognition, (2) novel methods tailored to examine phenotypes that emerge from clinical discovery, and (3) reverse-translated tasks used with human participants to optimize coordinated treatment development^5^.

Improvements in electrophysiological recording techniques for rodent subjects have also occurred in recent years and yielded significant advances in our understanding of neural mechanisms relevant to the study and treatment of neuropsychiatric disorders^6–8^. Given long-standing interest in characterizing rodent neurophysiological activity in response to different aspects of behavioral task performance^9–16^, it is surprising that developments in electrophysiology have largely proceeded independently from those in touchscreen-based models of animal cognition. However, several notable examples provide encouraging evidence of the value and feasibility of this unified approach. In mice, examples include engaging in a touchscreen-based visual discrimination and reversal learning while recording dorsal striatum neuronal activity^17^, a discrimination reversal task while recording single unit neuronal firing and oscillatory activity in the orbitofrontal cortex and dorsal striatum^18–20^, and a continuous performance task while recording prefrontal cortical–hippocampal oscillatory synchrony^21^. In rats, examples include engaging in a touchscreen-based object-cued spatial choice task while recording firing rates in the perirhinal cortex^22^ and a continuous performance task while recording medial prefrontal cortex, anterior cingulate cortex, prelimbic cortex, and infralimbic cortex activity^23^. Further confirmation of the ability to reliably capture key electrophysiological endpoints during a cognitive task within a touchscreen environment would advance the availability of innovative and diverse tasks for use in a highly translational platform, improving the alignment of psychiatry and neuroscience.

The present studies were designed to develop and optimize a touchscreen-based flanker task suitable for concurrent electrophysiological recordings in rats. In this commonly used computerized task to examine cognitive control in humans^24^, participants are instructed to quickly make one of two responses depending on a target stimulus presented in the center of the screen (e.g., if < is presented, press response key “left”; if > is presented, press response key “right”). During test sessions, the centered target stimulus is flanked on the left and right, either with congruent stimuli (e.g., <  <  <  < <) or incongruent stimuli (e.g., >  >  <  > >). Flanker stimuli are often briefly presented first and then joined by target stimulus presentation. The prepotent nature of the flanker stimuli causes interference effects evident in the loss of stimulus control from the centered target stimulus when flanked by incongruent flankers, as manifested by lower accuracy during incongruent relative to congruent trial types. Such interference effects are observed in healthy participants but are exacerbated, relative to heathy controls, in subjects with neuropsychiatric conditions characterized by deficits in cognitive control, including attention deficit hyperactivity disorder^25,26^, bipolar disorder^27,28^, depression^29^, Parkinson’s disease^30^, post-traumatic stress disorder^31^, and substance use disorders^32–34^.

The aim of developing a flanker task in rats to pair with electrophysiological recordings was chosen for several reasons. First, given repeated and successful pairing of the flanker task with electroencephalogram (EEG) recordings in human studies of cognitive control^35–40^, its reverse translation to laboratory animals could be a valuable tool for preclinical therapeutics development. Second, despite its frequent use in human studies of cognitive control, a rodent flanker task with comprehensive similarities to human protocols is not available (however, see^23,41–44^ for innovative rodent flanker task analogs). Third, the use of touchscreen apparatus enables precise control of various dimensions of visual stimuli to facilitate stimulus control^45,46^, which is critical for both target and flanker stimuli in rats given their notoriously poor visual acuity. Therefore, the present studies first evaluated the ability of rats to discriminate a variety of touchscreen-based visual stimuli (Experiment 1A) to subsequently serve in test sessions with congruent and incongruent flankers (Experiment 1B). Following task optimization, rats were implanted with electrophysiological probes (EEG skull screws and a local field potential [LFP] electrode) and neural responses to touchscreen-based visual evoked potentials (VEPs) following flanker and target stimulus onset were characterized (Experiment 2).

## Methods

### Subjects

Forty-one adult Long-Evans rats (36 male, 5 female) were used for the present studies; initial feasibility experiments involved males only, whereas subsequent experiments involved both sexes. Rats obtained from Charles River Laboratories (Wilmington, MA) weighing between 225 and 325 g were housed 3 to a cage, with the exception of those in Experiment 2 which were singly housed following surgical probe implantation, in a climate-controlled vivarium with a 12-h light/dark cycle (lights on at 7 am). In their home cage, access to water was unrestricted and access to food was restricted to daily post-session portions of ~ 10 to 15 g of rodent chow per subject. This daily allotment was based on previous studies^5,46^, which verified this level of restriction maintains sweetened condensed milk as a reinforcer across an extended number of daily sessions. Rats were randomly allocated to their respective experimental conditions under protocols that were approved by the Institutional Animal Care and Use Committee at McLean Hospital and carried out in accordance with the ARRIVE guidelines and the Committee on Care and Use of Laboratory Animals of the Institute of Laboratory Animals Resources, Commission on Life Sciences^47^.

### Apparatus

Schematics of the rat touch-sensitive experimental chamber have been published previously^5^. Briefly, a Plexiglas chamber (25 × 30 × 35 cm) was situated in a sound- and light-attenuating enclosure (40 × 60 × 45 cm). A 17″ touch-sensitive screen (1739L, ELO TouchSystems, Menlo Park, CA) comprised the inside right-hand wall of the chamber. An infusion pump (PHM-100-5, Med Associates, St. Albans, VT) outside the enclosure was used to deliver sweetened condensed milk solution (Sysco Corporation, Houston, TX) into the shallow reservoir (diameter: 3 cm) of a custom-designed aluminum receptacle (4 × 5 × 1 cm) that was mounted 2 cm above the floor bars and centered on the left-hand inside wall. A speaker bar (NQ576AT, Hewlett-Packard, Palo Alto, CA) mounted above the touchscreen was used to emit audible feedback. All experimental events and data collection were programmed in E-Prime Professional 2.0 (Psychology Software Tools, Inc., Sharpsburg, PA).

### Touchscreen response shaping

Modified response-shaping techniques were used to train rats to engage with the touchscreen^48^. A 5 × 5 cm blue square on a black background served as a response box and was centered on the touchscreen with its lower edge 10 cm above the floor bars. This required each rat to rear on its hind legs to make a touchscreen response with its paw. Each response was reinforced with 0.15 ml of 30% sweetened condensed milk, paired with an 880-ms yellow screen flash and a 440 Hz tone, and followed by a 5-s blackout period. Following reliable responding, the position of the response box was alternated 5 cm left and right of center across 100-trial training sessions. After responses with latencies < 5 s were reliably observed to each position, discrimination training commenced.

## EXPERIMENT 1A: Examination of discriminative ability of visual stimuli in male rats

**Figure 1:**
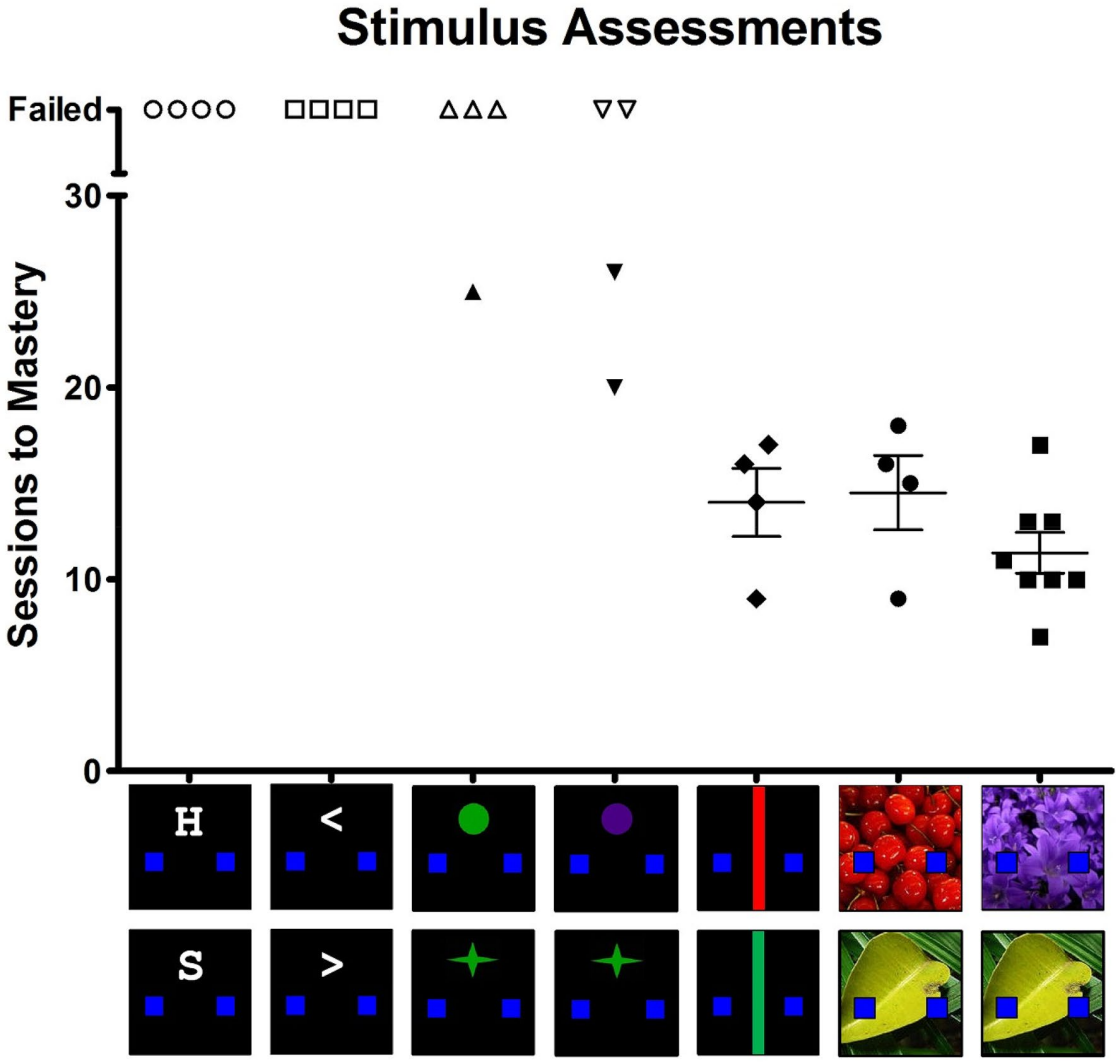
Discrimination training outcomes across touchscreen-based visual stimuli examined. Each data point represents the number of sessions required to meet ≥ 80% correct discrimination mastery criterion (or failure) for an individual subject and, when all subjects mastered the discrimination, horizontal lines represent group mean (± SEM).

The ability of rats to discriminate a variety of visual stimuli was first examined to identify those that could be most effectively and efficiently used in the touchscreen-based flanker task. Figure 1 depicts the stimuli assessed and their position on the screen relative to the blue response boxes. Table 1 details the size (cm) and illuminance (lux) of each stimulus. To capture chamber-wide illuminance values, the photo detector of a light meter (Model LT45, Extech Instruments, Nashua, NH) was positioned in the center of the chamber and perpendicular to the touchscreen, 15 cm from the screen and 5 cm above the floor bars (i.e., approximate rat eye level). Discrimination between two letters (H and S, *n* = 4) was evaluated because these letters were used as stimuli in the original flanker study^24^ and numerous subsequent experiments in humans^37,49,50^. Left and right arrows (< and >, *n* = 4) were examined because these stimuli are also frequently used in flanker studies with human participants^51–53^. Assessing whether two different green geometric shapes (green circle and green star, *n* = 4) could be discriminated was examined because one of the 2 types of retinal cone cells in the eye of Long-Evans rats^54^ has peak sensitivity at wavelengths approximating 510 nm^55,56^, which appears as a green hue. Assessing the ability of rats to discriminate between 2 different geometric shapes that also differed in color (purple circle and green star, *n* = 4) was examined because the second type of cone cell in Long-Evans rats displays peak sensitivity at wavelengths approximating 358 nm^57,58^. Although this second wavelength is in the ultraviolet portion of the color spectrum, purple is the closest visible color on the spectrum that can be presented on a standard touchscreen monitor. Vertical stripes (red stripe and green stripe, *n* = 4) were studied given their ability to span from the bottom to the top of the touchscreen and allow for flanking on the left and right. Finally, to evaluate whether complex photographic stimuli might provide additional colors, contrasts, and contours to assist the development of stimulus control, a predominately red and a predominately green photograph (red cherries and a green leaf, *n* = 4) or a predominately purple and a predominately green photograph (purple flowers and a green leaf, *n* = 8) were studied. Each stimulus pair described above was studied in a different group of male rats that were otherwise experimentally-naïve. All stimuli are available from the corresponding author upon request.

**Table 1:** Size (cm) and illuminance (lux) values for all stimulus arrangements examined in Experiments 1 and 2.

Stimulus	Size (cm)	Illuminance (lux)
White H	9 × 9.5	2.1
White S	9 × 9.5	2.1
White <	9 × 9	2.2
White >	9 × 9	2.2
Green circle	9 × 9	2.2
Green star	10 × 9	1.6
Purple circle	9 × 9	1.6
Red stripe	5 × 27	7.2
Green stripe	5 × 27	12.9
Red stripe flanked by red stripe	15 × 27	15.1
Red stripe flanked by green stripe	15 × 27	23.3
Green stripe flanked by green stripe	15 × 27	28.3
Green stripe flanked by red stripe	15 × 27	20.5
Red cherries (full screen)	34 × 27	9.3
Green leaf (full screen)	34 × 27	41.1
Purple flowers (full screen)	34 × 27	12.1
Red cherries (terminal size)	10 × 10	1.7
Green leaf (terminal size)	10 × 10	2.3
Purple flowers (terminal size)	10 × 10	2.0
Red cherries flanked by red cherries	33 × 10	2.1
Red cherries flanked by green leaf	33 × 10	2.8
Green leaf flanked by green leaf	33 × 10	3.3
Green leaf flanked by red cherries	33 × 10	2.6
Purple flowers flanked by purple flowers	33 × 10	2.5
Purple flowers flanked by green leaf	33 × 10	3.1
Green leaf flanked by purple flowers	33 × 10	2.7
Purple flowers flanked by 50% purple flowers	28 × 10	2.1
Purple flowers flanked by 50% green leaf	28 × 10	2.2
Leaf flanked by 50% leaf	28 × 10	2.5
Green leaf flanked by 50% purple flowers	28 × 10	2.4

Daily discrimination training sessions began with presentation of one of the two stimuli on the touchscreen (Fig. 1) and varied in a quasi-random manner across 100-trial sessions, with the provisos that there were exactly 50 trials of each stimulus type, and a given trial type would not be presented more than 5 times in a row. Subjects were trained to respond on the left or right 5 × 5 cm blue response box depending on the stimulus presented. A correct response was reinforced with 0.15 mL of 30% sweetened condensed milk delivered over 880 ms. Reinforcement was paired with the simultaneous presentation of a yellow screen and a 440 Hz tone and was followed by a 5-s blackout period. An incorrect response immediately resulted in a 10-s blackout period. Response box assignment was counterbalanced across subjects. To aid discrimination training, a correction procedure was implemented in which each incorrect trial was repeated until a correct response was made^59^. Because the primary objective of Experiment 1A was to determine which stimuli could be effectively and efficiently discriminated, each stimulus type was studied for a maximum of 30 sessions. The criterion for discrimination mastery was ≥ 80% correct (i.e., ≤ 10 repeats of each trial type per session) for 2 consecutive sessions.

## EXPERIMENT 1B: Examination of flanker congruency effects in male rats

**Figure 2:**
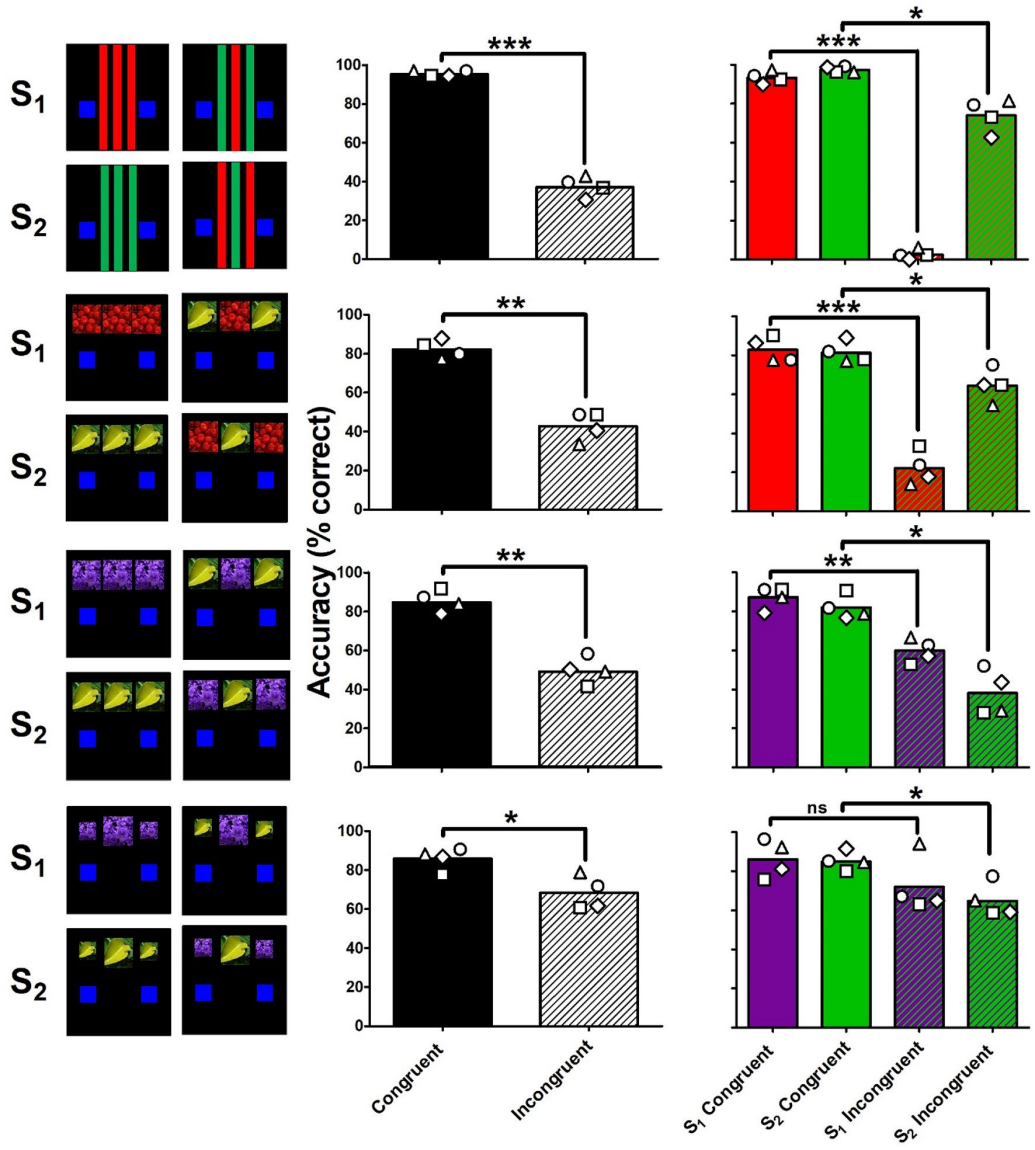
Congruency outcomes during flanker test sessions when using red/green stripes (top row), red cherries/green leaf (second row), purple flowers/green leaf (third row), purple flowers/green leaf with 50%-sized flankers (bottom row). Flanker depictions are shown in the left column, overall performance accuracy (% correct) during congruent/incongruent trials are shown in the middle column, and performance accuracy (% correct) during the 4 congruent/incongruent trial types are shown in the right column. Each data point in the middle and right columns represent performance from an individual subject.

The stimulus pairs that were discriminated to mastery criterion in all rats tested were advanced for subsequent examination in a touchscreen-based flanker task. In accord with standard human task protocols, 100-trial flanker test sessions were comprised of two thirds congruent trials (67/100) in which the centered target stimulus was flanked on the left and right with the same stimulus, interspersed with one third incongruent trials (33/100) in which the centered target stimulus was flanked on the left and right by the other stimulus. For the purple flowers/green leaf stimuli, flankers sized 50% of the target stimulus were also studied to determine whether this might increase accuracies during incongruent trial types relative to those obtained with full-sized flankers. Flanker stimuli appeared first, for 1 s, prior to presentation of the centered target stimulus. Thereafter, the left-flanker/centered-target/right-flanker stimulus array appeared on the screen until a response was made (see Fig. 2 for visual depictions of the 4 flanker trial types and Table 1 for the size (cm) and illuminance (lux) of the each 3-stimulus array). A correct response in each trial type was determined by an appropriate touch to the response box based on the centered target stimulus and was reinforced as described above. Three 100-trial flanker test sessions were conducted. Test sessions using the red and green stripes were conducted following at least 5 sessions of ≥ 80% correct on each trial type without the correction procedure implemented. For the red cherries/green leaf and purple flowers/green leaf stimuli, a 5-stage stimulus-fading procedure was used such that, initially, the photographic stimuli comprised the entire 17″ touchscreen, with response boxes superimposed in their positions described above. After ≥ 80% correct discrimination accuracy was observed on two consecutive sessions, the stimuli were made smaller by approximately 5 cm^2^ per stage. The correction procedure described above was only used during the first two training stages. This fading procedure was designed to efficiently train discriminative behavior in rats, while reducing the size of the stimuli to 10 × 10 cm to permit sufficient screen space on the left and right of the stimuli for congruent or incongruent flanking stimuli.

## EXPERIMENT 2: Electrophysiological responses during a flanker task in male and female rats

Following empirical validation of the purple flowers/green leaf stimuli with 50%-sized flankers in Experiments 1A and 1B, another group of rats (*n* = 9) was trained to discriminate between the two photographic stimuli exactly as described above with one exception. To avoid visually-evoked potentials from the yellow screen flash that was paired with reinforcement following correct responses in Experiment 1, two auditory tones (5 kHz and 15 kHz) were used during discrimination training and flanker testing in Experiment 2 to signal correct and incorrect responses (frequencies associated with correct and incorrect responses were counterbalanced across subjects). Although only male rats were studied in Experiment 1, both male (*n* = 4) and female (*n* = 5) rats were studied in Experiment 2. This allowed us to determine whether similar performance outcomes would be observed in both sexes under terminal conditions which also included electrophysiological recordings.

### Electrode implantation

Following discrimination training, rats underwent stereotaxic surgery to implant recording electrodes. Rats were anesthetized with isoflurane (1.5%) and skull screw EEG electrodes with a head diameter of 2.5 mm were lowered to dura over an occipital site (AP: − 7.0, ML: − 3.5), bilaterally at a frontal site (AP: + 3.7, ML: ± 2.6) and two cerebellar sites which served as reference and ground electrodes. In addition, a 0.1 mm diameter stainless-steel wire electrode was implanted unilaterally into the primary visual cortex (V1; AP: − 7.0, ML: + 3.5, DV: − 1.6) contralateral to the occipital screw to record LFPs. The electrodes were interfaced with an EIB-16 electrode interface board (Neuralynx) and all electrodes and the board were secured to the skull using dental acrylic. Following surgery, rats were singly housed and given a 7-day recovery period.

### Flanker task testing with concurrent electrophysiological recording

Following recovery from surgery, rats were given additional discrimination training sessions to re-establish discriminative performance to criterion. These post-surgery training sessions were conducted in an electrophysiological recording environment that consisted of identical touchscreen chambers inside a grounded faraday enclosure (ENV-018MD-EMS, Med Associates, St. Albans, VT). On select post-surgery training sessions, a head stage cable was connected to an RHD 16 channel amplifier board and secured, via an omnetics connector, to the head-mounted electrode interface board (Intan Technologies, Los Angeles, CA). Rats were then placed in the operant chamber and the head stage cable was attached to a commutator held in place above the chamber by a balance arm (PHM-110P1, Med Associates, St. Albans, VT) to allow unrestricted movement inside the chamber. Once discriminative performance to criterion was re-established with the head stage cable connected, rats were exposed to a flanker test session while concurrent electrophysiological recordings were conducted. Flanker test sessions consisted of 300 trials, with two-thirds (200/300) congruent and one-third (100/300) incongruent trial types.

### In vivo electrophysiological recording and data acquisition/reduction

Continuous EEG and LFP measurements were acquired during the flanker test session using the RHD-2000 recording system and supported data acquisition software (Intan Technologies, Los Angeles, CA). Signals were locally digitized via a 16-channel headstage and continuously sampled at 1 kHz with a bandwidth range of 0.1–300 Hz for the duration of the behavioral session. Signal analyses were performed using BrainVision Analyzer 2.0 (Brain Products, Gilching, Germany). Data were down-sampled offline to 500 kHz, bandpass filtered between 0.1 and 30 Hz and referenced offline to a common screw electrode placed in the cerebellum. Data obtained using electrodes with poor signal quality and movement artifacts were automatically rejected using a minimal-maximal allowed amplitude of − 300 µV and 300 µV, respectively, 200 ms before and after the event. For the VEP analyses, the signal from the visual and frontal electrodes was time-locked to the flanker stimulus onset for all trials using a pre-stimulus average amplitude baseline correction from − 500 to 0 ms. Only trials that passed the artifact rejection process were included in these analyses; however, very few trials were rejected (see Table 2). Of the 9 rats that were implanted with electrodes, one showed poor signal quality from the V1 LFP wire, so data from that electrode were not utilized for that rat. Thus, the final sample used for the VEPs analysis was 9 rats for the occipital and frontal screws (5 females, 4 males) and 8 rats for the V1 LFP wire (4 females, 4 males).

**Table 2:** Number of trials (out of 300) used to calculate the average VEPs for all the electrodes after the artifact rejection process.

Electrode	Rat 1	Rat 2	Rat 3	Rat 4	Rat 5	Rat 6	Rat 7	Rat 8	Rat 9	Mean
Left frontal EEG	299	277	297	300	274	286	298	300	246	290
Right frontal EEG	299	279	299	299	273	286	297	273	246	290
Occipital EEG	300	278	299	300	274	284	300	298	246	291
V1-LFP	191	267	292	225	246	282	251	221	N/A	251

### VEP internal reliability assessment

To determine whether the flanker-locked VEPs were consistent across trials, the internal reliability of the different amplitude values between 100 and 200 ms was calculated. This time window was selected because the peak amplitudes were observed at that time point. A split‐half reliability method was conducted to examine the correlation between odd‐ and even‐numbered trials^60^ adjusted by the Spearman–Brown (SB) correction formula: (SB = 2r/[1 + r]). Based on previously published reports^61–63^, a Spearman–Brown coefficient of 0.7 was considered as acceptable for internal reliability.

### Histology

**Figure 3:**
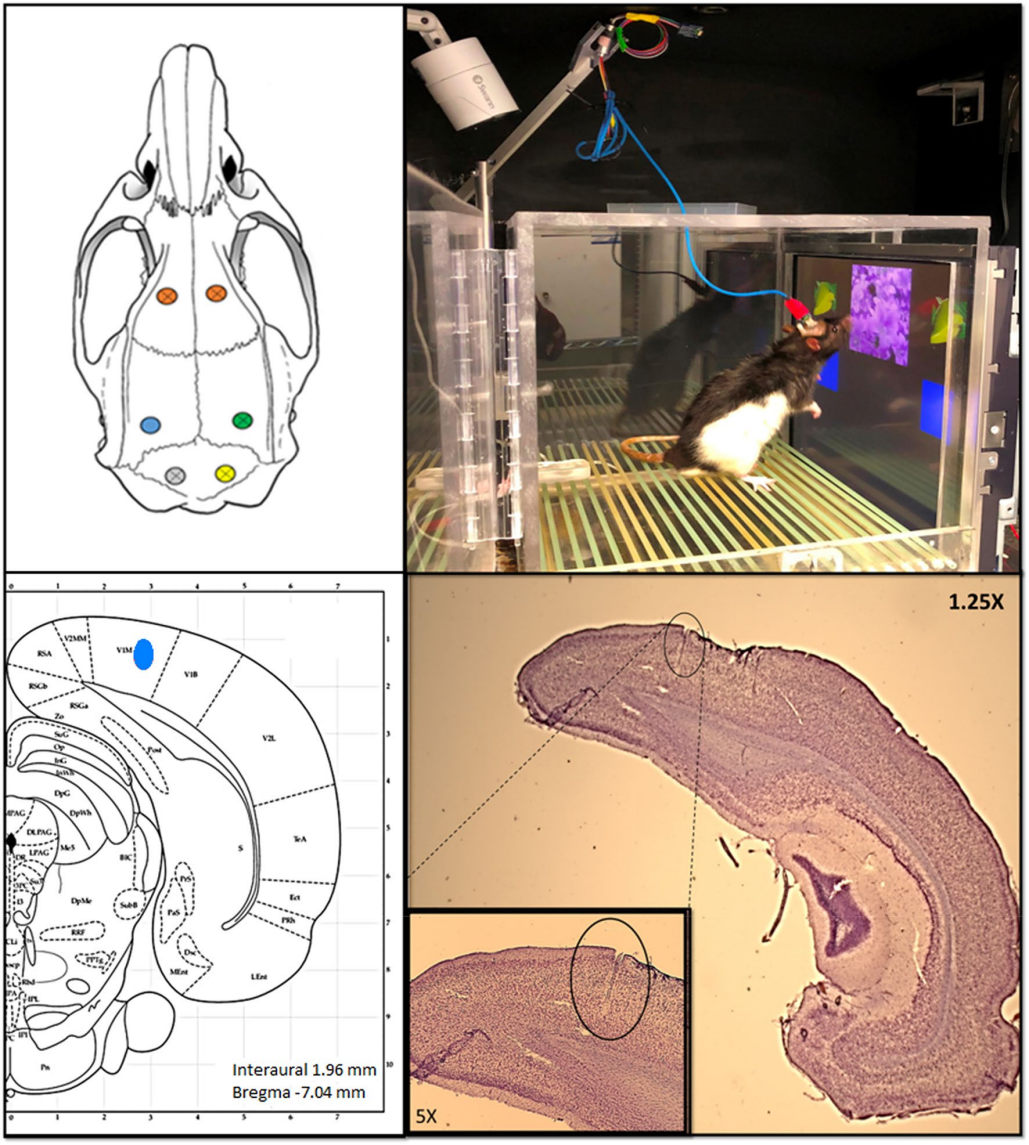
Upper left panel: electrode locations on rat skull (orange: bilateral screws on frontal region; blue: LFP wire in V1 area; green: screw on occipital region; yellow: ground screw; gray: reference screw). Upper right panel: photograph of the behavioral/recording setup. Bottom left panel: representative location of the V1 LFP wire on a rat brain atlas. Bottom right panel: representative histology image showing the LFP electrode placement in V1.

Upon completion of all test sessions, rats were euthanized, and brains were removed and fixed in 4% paraformaldehyde for at least 14 days. Brains were then sliced with a microtome into 30 µm sections, mounted, stained with cresyl violet, and cover slipped. Atlases showing recording locations and a representative placement for the V1 LFP wire are presented in Fig. 3.

### Statistical analyses

In Experiment 1A, sessions-to-mastery data from stimuli in which all subjects learned the discrimination to criterion were subjected to a one-way analysis of variance followed by a partial eta squared measure of effect size to statistically examine differences in acquisition rate. In Experiment 1B, paired t tests followed by Cohen’s *d* measure of effect size were performed to provide statistical analysis of flanker congruency effects among the visual stimuli studied, both by trial type and between S_1_ and S_2_ stimulus configurations. In Experiment 2, to determine possible differences in the visual response based on the stimulus color, a paired t test analysis was performed to compare the peak VEP amplitude values for all purple flowers and green leaf trials for the visual electrodes (i.e., V1 LFP: 100–200 ms; occipital screw: 75–125 ms). Statistical analyses were performed using GraphPad Prism 8 Software (San Diego, CA).

## RESULTS

### EXPERIMENT 1A: Examination of discriminative ability of visual stimuli in male rats

Figure 1 summarizes discrimination training outcomes across the touchscreen-based visual stimuli examined. Each data point represents the outcome for an individual subject and, when all subjects successfully mastered the discrimination, horizontal lines represent the group mean (± SEM). As the figure shows, none of the rats exposed to letter or arrow stimuli were able to meet discrimination mastery criterion, with performance in all subjects approximating chance throughout the 30-session condition. One subject was able to master the green shape discrimination following 25 sessions and 2 of 4 subjects were able to master the purple/green shape discrimination following 20 and 26 sessions; however, although the remaining subjects intermittently displayed performance above chance, none reached discrimination mastery criterion within the 30-session condition. All subjects reached discrimination mastery criterion at a rate that did not differ statistically (*F*[2,15] = 1.51, *p* = 0.26; η_p_^2^ = 0.188) when trained with the red/green stripes (mean 14.0, range 9–17), red cherries/green leaf (mean 14.3, range 8–18), and purple flowers/green leaf (mean 11.4 range 7–17).

### EXPERIMENT 1B: Examination of flanker congruency effects in male rats

Figure 2 summarizes congruency outcomes during the three 100-trial flanker test sessions comprising 2/3 congruent and 1/3 incongruent trial types. As the top row of panels shows, the expected congruency effects were observed in subjects trained and tested with the red/green stripes, such that accuracy during congruent flanker trial types using both color stripes were high, whereas incongruent flanker trial types were associated with low accuracy (*t*[3] = 28.85, *p* > 0.0001; *d* = 14.43). However, examining performance during each trial type revealed that this congruency effect was disproportionately driven by red stripe target trials (*t*[3] = 167.45, *p* < 0.0001; *d* = 83.73), with near-0% accuracy in all subjects during trials in which the red stripe target stimulus was flanked by green stripes, relative to congruency effects during green stripe target trials (*t*[3] = 5.16, *p* = 0.014; *d* = 2.58). As shown in the second row of Fig. 2, similar findings were observed in subjects trained and tested with the red cherries/green leaf stimuli (*t*[3] = 10.71, *p* = 0.002; *d* = 5.36); the congruency effect was largely driven by red cherries target trials (*t*[3] = 18.27, *p* = 0.0004; *d* = 9.14) relative to the green leaf target trials (*t*[3] = 4.07, *p* = 0.027; *d* = 2.04), although subjects were able to perform with somewhat higher accuracies when the red cherries target was flanked by green leaf stimuli (~ 22% correct). Replacing the red image with a purple image—thereby improving the match between the stimuli colors and the sensitivity of the 2 types of cone cells in the rat eye—dramatically improved performance: prototypical flanker congruency effects were observed in subjects trained and tested with purple flowers/green leaf stimuli. As shown in the third row of panels in Fig. 2, accuracy was, on average, 85% correct during congruent trial types and 49% correct during incongruent trial types (*t*[3] = 7.05, *p* = 0.006; *d* = 3.53). Although not as disparate as performance using red and green stimuli, congruency effects were also imbalanced when examining purple flowers target trials (*t*[3] = 5.70, *p* = 0.011; *d* = 2.85) and green leaf target trials (*t*[3] = 6.83, *p* = 0.006; *d* = 3.42). The purple flowers flanked by the green leaf trial type yielded on average 60% correct, whereas the green leaf flanked by purple flowers trial type was 38% correct. However, in subjects trained with purple flowers/green leaf and tested with flankers sized 50% of the target stimulus, accuracy during the congruent trial types remained on average at 85% correct, whereas incongruent trial types increased to 68% correct (*t*[3] = 5.40, *p* = 0.012; *d* = 2.70), a product of two incongruent trial types with improved balance (72% and 65% correct) in interference effect (*t*[3] = 2.20, *p* = 0.115; *d* = 1.10; *t*[3] = 4.03, *p* = 0.027; *d* = 2.02, respectively). Reaction time measures across visual stimuli and congruency trial types tested were highly similar across subjects, approximating on average 2–3 s following target stimulus onset (data not shown).

### EXPERIMENT 2: Electrophysiological responses during a flanker task in male and female rats

**Figure 4:**
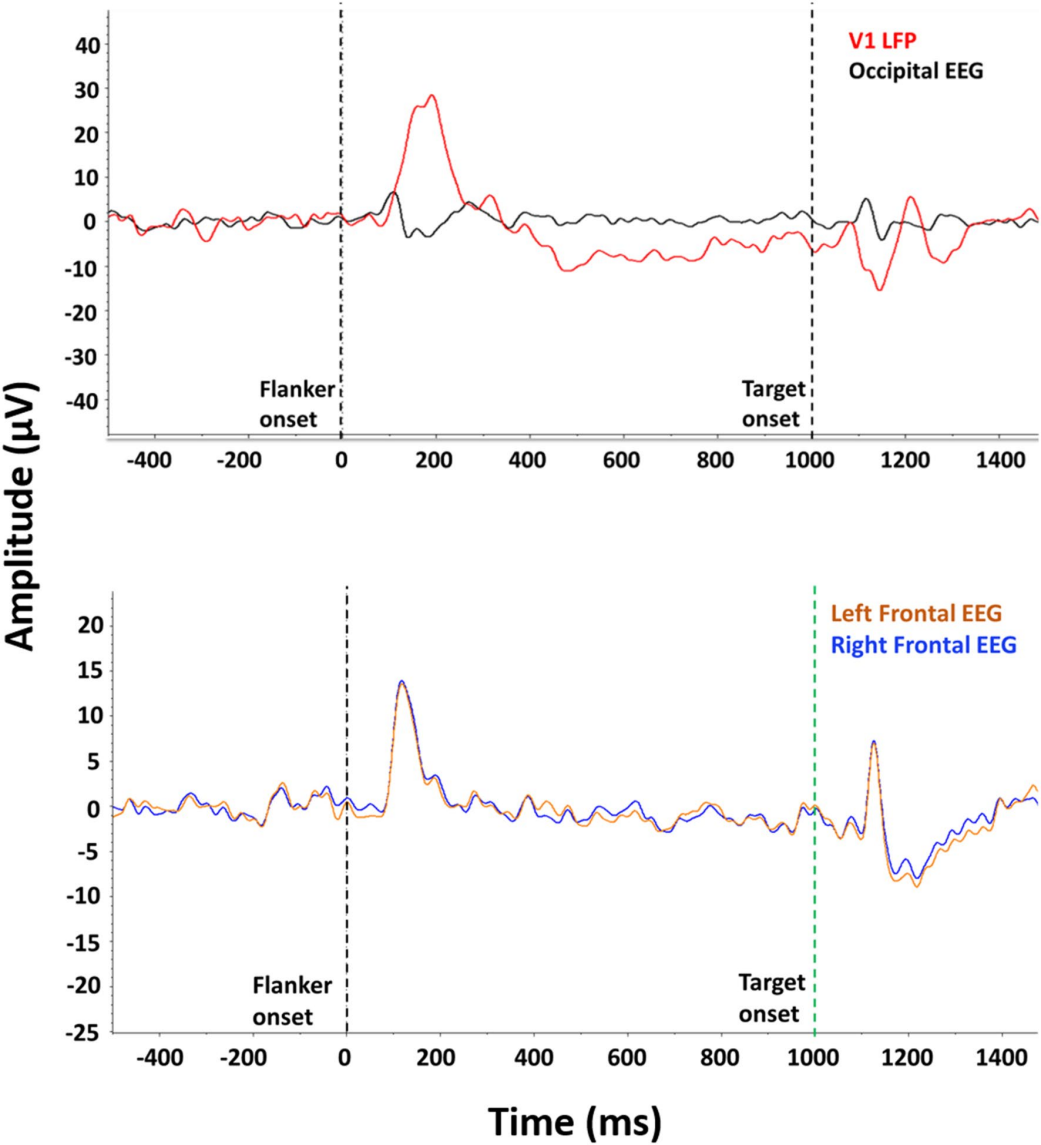
Flanker-locked VEPs for electrodes located in visual and frontal areas. Top panel: VEP grand average of all trials for the occipital EEG electrode (black tracing) and the V1 LFP electrode (red tracing). Bottom panel: VEP grand average of all trials for the left (orange tracing) and right (blue tracing) EEG electrodes. First vertical dashed line (time zero) corresponds to the flanker stimulus presentation and second vertical dashed line (at 1000 ms) represents the target stimulus onset. V1 LFP wire, *n* = 8; occipital and frontal screws *n* = 9.

The 9 subjects in Experiment 2 reached discrimination mastery criterion following an average of 14.11 (± 1.86) sessions, which was comparable to subjects trained with the purple flowers/green leaf stimuli in Experiment 1A. Following electrode implantation, subjects had a 7-day recovery period followed by additional discrimination training until mastery was again observed following the surgical procedure. The recovery of criterion levels of discrimination mastery after surgery prior to flanker test sessions took an average of 33.22 (± 4.15) sessions. Task performance during the 300-trial flanker test session was also similar to values observed in Experiment 1B, with 82% congruent and 65% incongruent group-mean accuracies (*t*[8] = 5.49, *p* = 0.0006; *d* = 1.83). Congruency outcomes were similar between sexes, but the females showed nominally higher levels of accuracy during both trial types (85% vs. 77% congruent; 66% vs. 63% incongruent). However, considering the relatively small sample size, it is presently uncertain if this is a meaningful sex difference. To identify neurophysiological signatures of processes elicited by touchscreen-based visual stimulus presentation, flanker-locked VEP analyses were conducted for the visual (V1 LFP wire and occipital screw) and frontal electrodes (left and right frontal screws) during the 300-trial flanker test session. As shown in the top panel of Fig. 4, flanker stimulus presentation generated a VEP on the occipital EEG electrode (black tracing) with a positive peak at 100 ms followed by a negative amplitude deflection around 140–200 ms; whereas the V1 LFP channel (red tracing) showed an inverse polarity during this time window with a positive peak at 200 ms. In the V1 LFP channel, a negative amplitude deflection approximating 1150 ms followed by a positive peak at 1200 ms (i.e., 150 and 200 ms after target stimulus presentation, respectively) was also observed. In addition, a small positive peak around 1100 ms (i.e., 100 ms after target stimulus presentation) was observed in the occipital EEG channel, which was highly similar to the positive peak observed after the flanker stimulus. As shown in the bottom panel of Fig. 4, the left (orange tracing) and right (blue tracing) frontal EEG electrodes displayed a positive peak 100 ms after the flanker stimulus presentation was observed in both channels, another positive peak following approximately 1100 ms, and, as well, a negative amplitude deflection around 1200 ms, which corresponds to 100 ms and 200 ms after the target presentation, respectively.

**Figure 5:**
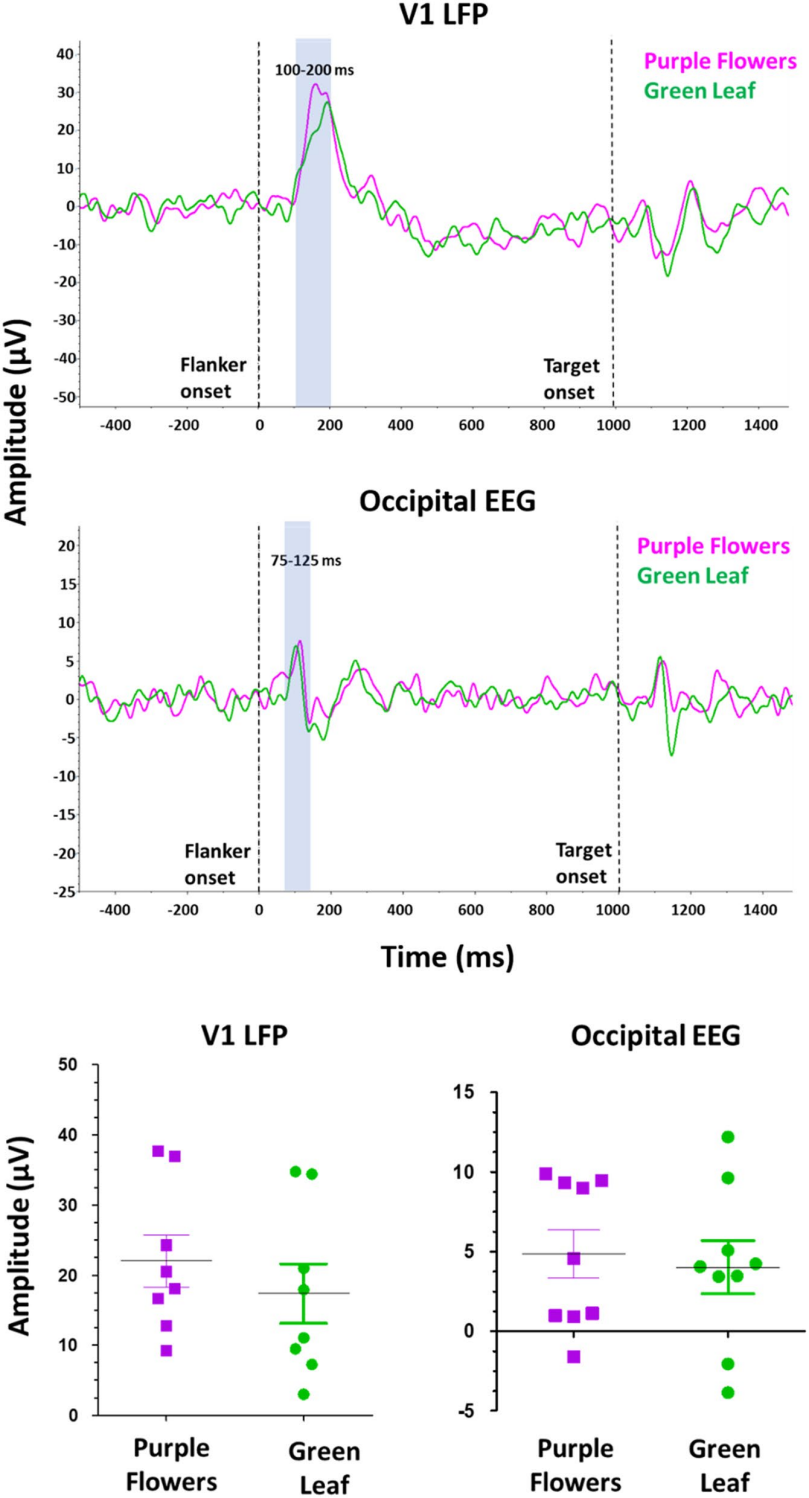
Stimulus–response comparison of VEPs for electrodes located in visual areas by trial type. Top panel: V1 LFP electrode. Middle panel: VEPs of the occipital EEG. Bottom panels: average amplitude of the V1 LFP electrode at the 100–200 ms time window (left) and occipital EEG at the 75–125 ms time window (right). First vertical dashed line (time zero) corresponds to the flanker stimulus presentation and second vertical dashed line (at 1000 ms) represents the target stimulus onset.

Figure 5 presents findings during purple flowers (purple tracing) and green leaf (green tracing) trial types from the V1 LFP (top panel), occipital electrode (middle panel), and average individual-subject amplitudes for V1 LFP (bottom-left panel) and occipital (bottom-right panel) electrodes. Taken together, no significant differences were observed in the VEP peak amplitudes (V1 LEP: 100–200 ms; occipital screw: 75–125 ms) between purple flowers and green leaf flanker trial types in the visual electrodes (V1 LFP: *t*[7] = 1.90, *p* = 0.10; *d* = 0.66; occipital screw: *t*[8] = 0.43, *p* = 0.68; *d* = 0.15).

**Figure 6:**
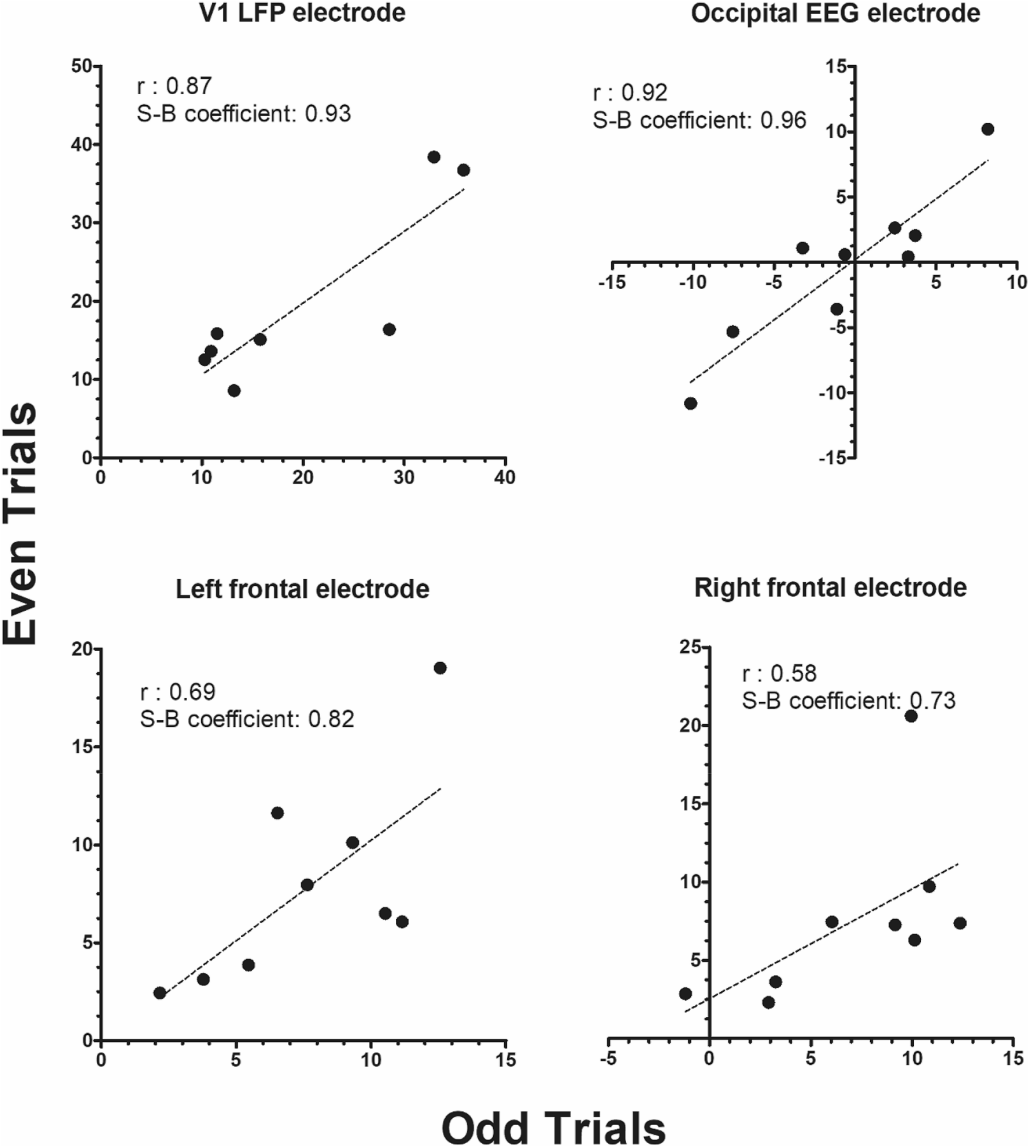
Internal reliability of VEPs across trials as split-half reliability for odd and even trials. Panels show average amplitude for odd and even trials time-locked to the flanker stimulus presentation across the 100–200 ms time window and the Pearson correlation (r) and Spearman–Brown (S–B) coefficient for the (**a**) V1-LFP electrode, (**b**) occipital EEG electrode, (**c**) left frontal electrode, and (**d**) right frontal electrode.

Figure 6 presents split-half internal reliability analysis of amplitude values in the 100–200 ms time window. As the figure highlights, high flanker-locked VEP reliability coefficients were observed for both visual electrode types (Spearman–Brown coefficients: 0.96 for occipital electrode; 0.93 for V1 LFP electrode) and for the left and right frontal EEG electrodes (Spearman–Brown coefficients: 0.82 for occipital electrode; 0.73 for V1 LFP electrode). These data indicate that the flanker task developed in Experiment 1 was capable of eliciting highly reliable VEP components across trials.

## Discussion

The first objective of the present studies was to optimize visual stimulus parameters and develop a touchscreen-based flanker task for rats with improved alignment to that used with human participants. Under the conditions tested in these studies, Long-Evans rats were unable to effectively discriminate between the visual stimuli most commonly used in human flanker tasks (i.e., left and right arrows or the letters H and S). In addition, some but not all rats were able to efficiently discriminate between green stimuli that differed in geometric shape (i.e., green circle and green star) or between different geometric shapes that differed in color (i.e., purple circle and green star). It is presently unclear why the rats were unable to master the letter and arrow stimuli as there are numerous instances in the extant literature of rats learning visual discriminations using white shapes imposed on black screen backgrounds^64^. It is conceivable that idiosyncratic features of these stimulus prevented mastery; for example, their size, contours, or placement on screen may not have been optimal. Given that some, but not all, subjects mastered the green and purple geometric shapes, and in view of their common use as rodent touchscreen stimuli, it seems likely that discrimination mastery may have been observed following extended training. However, these studies were intended to prioritize efficiency in acquisition rate, which did not occur with these stimuli.

All rats trained using red/green stripes, red cherries/green leaf, and purple flowers/green leaf were able to efficiently master these discriminations in fewer than 30 sessions. When using these stimulus pairs in a flanker test session, the expected flanker interference effects of lower accuracies during incongruent trials were observed using all arrangements; however, there were large disparities between the interference effects when using incongruent red- and green-colored flanking stimuli. Imbalanced interference effects were also observed using incongruent purple flowers and green leaf flanking stimuli, but to a much lesser extent. When flankers sized 50% of the target stimulus were used, minimal imbalance was observed and desirable psychometrics (in terms of congruent and incongruent accuracy scores) were achieved. This modification was also associated with increased accuracies during incongruent trial types that better approximated values in our previous work with human participants^29,63^. It is also important to note that differences in the relative sizes of target and flanker stimuli in rat studies represent a departure from the prototypical task variants used with human participants. However, we believe that functional equivalency is an important metric to align cross-species task versions.

Although directly probing the spectral mechanics of the rat visual system was outside the scope of the present studies, previous work detailing the frequency band tuning of the two cones in the Long-Evans rat^54^ provides important context for interpreting Experiment 1 findings. Rats possess two types of retinal cone cells, one of which has peak sensitivity at wavelengths approximating 510 nm^55,56^, which appears as a green hue; the other type of cone cell has peak sensitivity at wavelengths approximating 358 nm^57,58^, which is on the ultraviolet portion of the color spectrum. Therefore, given substantial distance of the red stripe and red cherries from peak sensitivity at either type of cone cell, limited salience likely accounts for the poor stimulus control when flanked by green stimuli. The purple flowers stimulus was expressly chosen because it was as close as possible to ultraviolet on the visible color spectrum using a standard touchscreen monitor. Interestingly, one might presume the green leaf would more actively stimulate the 510 nm cone cells when compared to the ability of the purple flowers to stimulate the 358 nm cone cells—considering the relative distances of these colors from the optimal tuning of the respective cone cells—and thus appear more salient to the rat. However, performance during incongruent trials using these stimuli indicates that was not the case. It is important to note that other stimulus dimensions, such as illuminance and contour, likely played key roles; however, they were not parametrically manipulated in the present studies. Nevertheless, from a task development perspective, it is notable that green stimuli presented on a touchscreen will not necessarily overshadow stimuli of other colors on the visible color spectrum.

Following flanker task optimization for rats, Experiment 2 provided key electrophysiology findings that support the objectives of concurrent cognitive testing and neurophysiological recordings. Electrophysiological signatures of the visual response associated with flanker and target stimulus onset during optimized task conditions were identified, both for the electrodes located in visual (Fig. 4, top panel) and frontal (Fig. 4, bottom panel) areas. Moreover, consistent with previous studies in behaving rats^65^ and anesthetized monkeys^66^, rat visual areas were also observed to be highly active 100–200 ms after the stimulus onset and generated a V1 LFP signal by feedback to V1, as in the visual electrodes (Fig. 4, top panel). Accordingly, these data also showed a small negative deflection in the occipital screw at the time of the largest positive V1 LFP signal (Fig. 4, top panel). A possible explanation is that the reversal of the polarity at the occipital screw could arise with variation of the depth profile across cortical layers^67^, which is also an indication that the neuronal processes differ at the time of the signal onset (~ 100 ms) and at the time of the peak (~ 200 ms). This apparent biphasic nature of the occipital screw signal could also explain why the amplitude was lower than for the frontal screws (Fig. 4, top panel and bottom panel).

Additional correspondence between neurophysiological and behavioral findings was evident in electrophysiological signatures following visual display of the purple flowers and green leaf stimuli selected for the optimized rat flanker task. In line with the relatively balanced behavioral interference effects in Experiment 1B, no significant differences across flanker and target stimulus trial types were observed in the VEPs for the electrodes located in visual areas (Fig. 5). Internal reliability calculations of the signal across trial types provided confirmation of consistent electrophysiological measures under optimized task conditions (Fig. 6), which should be predictive of robust and orderly replication using this approach in future studies.

Finally, although use of photographic stimuli is not as common in human flanker studies as arrow and letter stimuli, several studies have successfully used pictorial stimuli^68–72^. In addition, we have recently used these exact purple flowers and green leaf stimuli with human participants and found them to produce prototypical behavioral and electrophysiological flanker interference effects^63^. Moreover, between-species comparisons across homologous neuroanatomical loci in healthy human participants and rats using this protocol have revealed similar stimulus-locked increases in theta power during high-conflict trials as well as error-related negative potentials in recent studies^73^.

In conclusion, the present studies indicate that expected outcomes in measures of both brain wave activity and complex behavior can be obtained from a touchscreen-based cognitive task with concurrent electrophysiological recordings. As Experiment 1 highlights, parametric studies of visual stimuli help achieve desired goals, in the case of the present studies, reverse translation of a common human task with formal and functional similarity in task arrangement and performance outcomes. The balance in salience and stimulus control provided by purple flowers and green leaf target and flanker stimuli following optimization efforts in Experiment 1, was accompanied by well-balanced electrophysiological signatures between flanker task trial types, as reflected in Experiment 2 findings. Taken together, it is highly encouraging that these approaches can be effectively and efficiently combined within a complex touchscreen-based task. Advancements of this sort will allow for further development of novel and innovative multimodal platforms needed for coordinated cross-species preclinical therapeutic development.
